# Ferrotoxicity and Its Amelioration by Calcitriol in Cultured Renal Cells

**DOI:** 10.1155/2021/6634429

**Published:** 2021-02-22

**Authors:** Chandrashekar Annamalai, Rohit Seth, Pragasam Viswanathan

**Affiliations:** ^1^Renal Research Lab, Centre for Biomedical Research, School of Biosciences and Technology, Vellore Institute of Technology (VIT), Vellore, 632 014 Tamil Nadu, India; ^2^Department of Zoology, Guru Ghasidas Vishwavidyalaya, Bilaspur, 495009 Chhattisgarh, India

## Abstract

Globally, acute kidney injury (AKI) is associated with significant mortality and an enormous economic burden. Whereas iron is essential for metabolically active renal cells, it has the potential to cause renal cytotoxicity by promoting Fenton chemistry-based oxidative stress involving lipid peroxidation. In addition, 1,25-dihydroxyvitamin D3 (calcitriol), the active form of vitamin D, is reported to have an antioxidative role. In this study, we intended to demonstrate the impact of vitamin D on iron-mediated oxidant stress and cytotoxicity of Vero cells exposed to iohexol, a low osmolar iodine-containing contrast media *in vitro*. Cultured Vero cells were pretreated with 1,25-dihydroxyvitamin D3 dissolved in absolute ethanol (0.05%, 2.0 mM) at a dose of 1 mM for 6 hours. Subsequently, iohexol was added at a concentration of 100 mg iodine per mL and incubated for 3 hours. Total cellular iron content was analysed by a flame atomic absorption spectrophotometer at 372 nm. Lipid peroxidation was determined by TBARS (thiobarbituric acid reactive species) assay. Antioxidants including total thiol content were assessed by Ellman's method, catalase by colorimetric method, and superoxide dismutase (SOD) by nitroblue tetrazolium assay. The cells were stained with DAPI (4′,6-diamidino-2-phenylindole), and the cytotoxicity was evaluated by viability assay (MTT assay). The results indicated that iohexol exposure caused a significant increase of the total iron content in Vero cells. A concomitant increase of lipid peroxidation and decrease of total thiol protein levels, catalase, and superoxide dismutase activity were observed along with decreased cell viability in comparison with the controls. Furthermore, these changes were significantly reversed when the cells were pretreated with vitamin D prior to incubation with iohexol. Our findings of this *in vitro* model of iohexol-induced renotoxicity lend further support to the nephrotoxic potential of iron and underpin the possible clinical utility of vitamin D for the treatment and prevention of AKI.

## 1. Introduction

The prevalence of acute kidney injury (AKI) is increasing, and besides causing significant morbidity and mortality, it increases the risk of progression to irreversible kidney disease, thereby imposing a tremendous financial, societal, and personal burden [1, 2]. The pathogenesis of AKI is multifactorial and involves a complex [3] interaction between vascular, tubular, and inflammatory factors. This is followed either by repair and restoration of glomerular and tubular functions or culminates in fibrosis and progression to chronic kidney disease [4]. Currently, there is no specific and effective therapeutic modality and the treatment is mostly supportive in nature [5].

Furthermore, despite the incidence being overestimated, contrast-induced acute kidney injury (CI-AKI) still poses a major threat to patients undergoing contrast-associated transcatheter diagnostic and interventional procedures by worsening short- and long-term outcomes and prolonging hospital stay [6, 7]. Iohexol, a water-soluble, low osmolar, and nonionic iodine-containing monomeric radiocontrast agent induces renal damage by several mechanisms which are quite complex and yet to be completely understood [8, 9]. Renal hypoperfusion and renal medullary hypoxia [8, 10], autocrine and paracrine impairment including increased endothelin and adenosine release, lower nitric oxide metabolite concentration and enhanced oxidative stress [11] [12], and direct cellular damage [13] resulting in endothelial dysfunction followed by vasoconstriction of vasa recta [14] [15], and consequent alterations in renal blood flow lead to the development of contrast-induced renal injury. High viscosity can further adversely affect renal perfusion and urine formation [8].

Besides, the metallobiology of iron has been in focus in recent times due to its crucial pathophysiological role in kidney diseases. In particular, iron has been increasingly implicated in the induction of AKI and worse outcomes [16–20]. In this context, the cytotoxic potential of iron in contrast-induced AKI has been put forth as an important mechanism [18]. Iron is an indispensable component of oxygen-binding molecules (for example, hemoglobin and myoglobin), cytochromes in the electron transport chain, and as a cofactor in many enzymes and is involved in many fundamental biological processes [21, 22]. The kidneys play a vital role in preventing iron loss in urine by reabsorbing the filtered iron. Furthermore, they express several proteins necessary for iron transport and metabolic activity and are thus actively involved in systemic iron homeostasis [23, 24]. Although iron is essential for the metabolically active renal cells [25], it has the potential to induce renal cytotoxicity [26, 27]. This is attributed to the unique property of iron to mediate electron transfer and participate in the oxidation-reduction (redox) reactions. While this is crucial for the functioning of several biological systems, it can render iron lethal by catalysing the Fenton and Haber-Weiss reactions and promoting the production of reactive oxygen species (ROS) such as hydroxyl radicals. This further overrides the cellular antioxidant defence mechanisms and induces oxidative injury of cell structures in addition to causing local inflammation and vasoconstriction [26, 27]. It is worth noting that knowledge on the complex mechanisms of renal iron handling and iron-induced cellular injury (ferroptosis) is limited and its understanding could provide novel therapeutic avenues [22, 28].

Concurrently, the kidneys also possess endogenous and exogenous protective agents to counteract cellular injury [28]. In this context, vitamin D is known to have cytoprotective action because of its antioxidant potential [29–31]. Vitamin D is a prohormone with no intrinsic biological activity and is derived endogenously from the skin and exogenously from diet and supplements [32]. Both vitamin D2 (ergocalciferol) and vitamin D3 (cholecalciferol) differ in their side chains thereby affecting the capacity to bind to vitamin D-binding protein (DBP) as well as efficacy. Vitamin D3 is considerably more effective than vitamin D2 [33, 34]. During exposure to the sun, ultraviolet rays (270–300 nm) photolytically converts 7-dehydrocholesterol by breaking its B ring to form previtamin D3 which undergoes thermal isomerization to vitamin D3 [35, 36]. Vitamin D3 bound to DBP is sequentially hydroxylated first in the liver by the cytochrome P450s (microsomal CYP2R1 and mitochondrial CYP27A1) [37] to form 25-hydroxyvitamin D3 and subsequently in the proximal tubules of the kidney [38, 39] by 1 *α*-hydroxylase (CYP27B1) to the bioactive 1,25-dihydroxyvitamin D3, also known as calcitriol [40–42].

1,25-Dihydroxyvitamin D3 is known to possess several pleiotropic effects [43, 44]. Apart from being a key regulator of calcium homeostasis by modulating parathyroid hormone secretion and increasing gut calcium absorption [45], it has been proven to exert antioxidant [30, 31], anti-inflammatory, antiproliferative, and antineoplastic effects [46–49] as well. By virtue of these properties, calcitriol is cytoprotective by nature, whereas iron, especially the free form, is potentially cytotoxic. Furthermore, the role of vitamin D in AKI is not clearly elucidated as in chronic kidney disease [50] and it needs to be ascertained if vitamin D could mitigate ferrotoxicity induced by radiocontrast media *in vitro* [28].

Therefore, we sought to investigate the combined roles of iron and vitamin D in relation to oxidative stress and nephrotoxicity in an *in vitro* model of iohexol-induced AKI using Vero cells. The main purpose of this study was to determine the effect of iohexol on total cellular iron concentration and its influence on oxidative stress and cytotoxicity of Vero cells and to study the impact of 1,25-dihydroxyvitamin D3 on iron-mediated oxidant stress and cellular injury.

## 2. Materials and Methods

### 2.1. Cell Culture

Vero cells (ATCC® CLL-81™) were procured from Cell Repository, National Centre for Cell Science (NCCS), India. Dulbecco's Modified Eagle's Medium (HiMedia Laboratories, India) containing 10% fetal bovine serum (FBS) and 1% penicillin and streptomycin combination was used. The cells were grown on a cover glass in 10 cm plastic dishes placed inside an incubator at 37°C containing a humid atmosphere consisting of 5% oxygen, 5% carbon dioxide, and 90% nitrogen until a monolayer was formed. Periodic assessment of these cells was carried out to ensure freedom from mycoplasma contamination. Upon reaching 80-95% confluency, the cells were digested with trypsin, resuspended in serum-free medium, and passaged in a 1 : 3 proportion. The culture media was replaced every 2 to 3 days to ensure continuous nutritional support for cell growth.

The optimal dose of iohexol was determined and the cell viability (MTT (3-(4,5-dimethylthiazol-2-yl)-2,5-diphenyltetrazolium bromide)) assay was studied by plating 1 × 10^4^ viable cells per cm^2^ using 96-well plates.

Control cells were incubated similarly with sterile 0.9% normal saline (sodium chloride injection, B.P. 0.9% *w*/*v*, Schwitz Biotech, India) except that they were not pretreated with vitamin D3 and iohexol. Microscopy was performed using a Leica DM IL inverted fluorescent microscope equipped with appropriate fluorescent filters (Leica Microsystems, India). Images were captured at 100x magnification using an attached Leica DFC 450C camera and processed with LAS X software and exported.

### 2.2. Preparation of 1,25-Dihydroxyvitamin D3

1,25-Dihydroxyvitamin D3 (Cat: sc-202877, Santa Cruz Biotechnology Inc., USA) dissolved in absolute ethanol (0.05%, 2.0 mM, HiMedia Laboratories, India) was used for our cell culture studies. Vitamin D is light-sensitive and is stored away from direct light in a brown bottle at -20°C. On the day of use, further dilutions were made directly in the culture medium at the concentration of 1 *μ*M, a dose at which no significant change in cell viability was observed to occur.

### 2.3. Determination of the Optimal Dose of Iohexol

**Figure 1:**
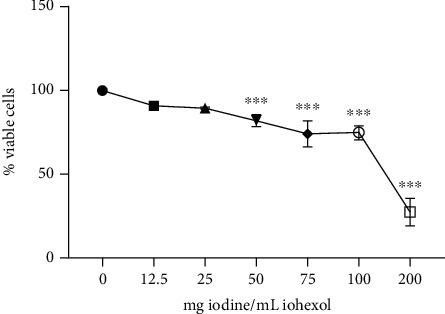
MTT 3-(4,5-dimethyl (thiazol-2-yl)-2,5-diphenyl tetrazolium bromide) assay to quantify cell viability in Vero cells exposed to graded concentrations of iohexol. One-way ANOVA and Tukey posttest (*n* = 4) were used to analyse the results. ^∗^*p* < 0.05, ^∗∗^*p* < 0.01, and ^∗∗∗^*p* < 0.001 (2-tailed) in comparison with the control.

Vero cells were treated with different concentrations of iohexol (OMNIPAQUE™, 350 mg iodine per mL, GE Healthcare, India) in a graded manner (12.5 mg/mL to 200 mg of iodine/mL) to determine the optimal dose at which significant cellular injury occurred as reflected by the characteristic morphological changes on microscopy in comparison with a control group. Cells exposed to iohexol show signs of rounding up. In contrast, the control healthy cells retain their primary elongated shape. The cell viability was further assessed by MTT assay as described below (Figure 1) by exposing the Vero cells to increasing doses of iohexol. An effective dose of 100 mg iodine per mL of iohexol was used for the successive experiments.

### 2.4. Determination of Cellular Iron by Atomic Absorption Spectroscopy

The concentrations of Fe in Vero cells were measured using a flame atomic absorption spectrophotometer (AAS) (Hitachi, Japan). Iron stock standard solution (1 g/L) was purchased from Sigma-Aldrich, USA, and nitric acid (99%) from HiMedia Laboratories, India. Standard solutions were prepared by diluting 1 g/L iron solution with 1.5% HN0_3_ solution at various concentrations. Samples were prepared at 10-fold dilution by diluting the homogenized cell samples with 1.5% HN0_3_ solution. Deionized distilled water was used as blank. Standard solution was then mixed with distilled water, and a calibration curve was generated to estimate the levels of iron in the deionized distilled water. Following this, 40 mL of the sample preparation was injected into the atomic absorption spectrometry [51–54] in order to quantify iron in the Vero cells.

### 2.5. Reactive Oxygen Species Production

Lipid peroxidation was determined by TBARS (thiobarbituric acid reactive species) assay by spectrophotometric method [55]. The antioxidant status was assessed by measuring the levels of total thiol protein using Ellman's method [56] and the activities of catalase by colorimetric method [57] and superoxide dismutase (SOD) by nitroblue tetrazolium assay [58].

### 2.6. Detection of Cell Toxicity Using DAPI

Control Vero cells resuspended with 200 *μ*L of 1 *μ*g/mL DAPI (4′,6-diamidino-2-phenylindole dihydrochloride; Cat: D9542, Sigma-Aldrich, USA) in phosphate-buffered saline (PBS) were incubated for five minutes at room temperature while rotating and then washed using PBS mixed with a solution of 0.1% sodium azide. 2 *μ*L of these cells was transferred to a coverslip coated with poly-l-lysine and left in the dark for five minutes. This coverslip was subsequently placed over a glass slide containing 5 *μ*L of antifade solution and left for another five minutes in the dark. The cells were finally examined under a fluorescent microscope [59, 60]. Similarly, Vero cells were pretreated with 1 *μ*M 1,25-dihydroxyvitamin D3 with and without 100 mg/mL iohexol and investigated further.

### 2.7. Assessment of Cell Viability by MTT Assay

An MTT assay was carried out to determine inhibition of cell activity and to quantify metabolically viable cells [61]. This is a colorimetric assay where the soluble yellow tetrazolium salt (MTT) is reduced to insoluble blue-purple formazan crystals [62] by the mitochondrial succinate dehydrogenase enzyme activity [63]. Vero cells in the logarithmic growth phase were inoculated at a seeding density of 1 × 10^4^ cells in to a 96-well plate. At around 80-90% confluence, varying concentrations of iohexol (12.5, 25, 50, 75, 100, and 200 mg iodine/mL) were added and left at room temperature (37°C) for 3 hours. Subsequently, 20 *μ*L (1 mg/mL) of MTT labelling reagent (HiMedia Laboratories, Mumbai, India) in sterile PBS was instilled in to each well followed by incubation for 4 hours at 37°C. The cells were then examined under a phase-contrast microscope to study the cell confluency. The insoluble, purple formazan crystals thus formed were dissolved by mixing the cells with 150 *μ*L of dimethyl sulfoxide and shaking the cell plate for 5 minutes to enable complete solubility. The cell viability of the treated cells was assessed by determining the optical density at 570 nm using a spectrometer (EPOCH-2 plate reader, BioTek Instruments, USA) and analysing the results in comparison with the control group which displayed 100 percent viability. The procedure was repeated in Vero cells pretreated with 1 *μ*M of 1,25-dihydroxyvitamin D3 dissolved in 2.0 mM of 0.05% absolute ethanol. All the experiments were performed four times [64, 65].

### 2.8. Experimental Protocol

Vero cells were treated with iohexol at a concentration of 100 mg/mL for 3 hours. The same stimulation was replicated after pretreatment with 1,25-dihydroxyvitamin D3 dissolved in absolute ethanol (0.05%, 2.0 mM) at a dose of 1 *μ*M for another 6 hours. Control cells were exposed to 0.9% normal saline. Cellular iron was determined by atomic absorption spectroscopy, oxidative stress was assessed by the methods described above, the cells were stained with DAPI (4′,6-diamidino-2-phenylindole), and the extent of cellular injury was assessed by the uptake of tetrazolium MTT dye by the control cells and the Vero cells exposed to iohexol before and after pretreatment with 1,25-dihydroxyvitamin D3.

### 2.9. Statistical Analysis

The experimental results from four independent experiments (technical replicates) were represented as means ± standard deviation (SD). One-way analysis of variance (ANOVA) with Tukey multiple comparison posttest was used to study the individual variances in relation to the control group. Pearson correlation was used to estimate the association between different study parameters. Statistical significance was defined at *p* < 0.05 (2-tailed). SPSS (IBM Corp. Released 2017, IBM SPSS Statistics for Windows, Version 25.0. Armonk, NY: IBM Corp.) was used to analyse the results and GraphPad Prism Version 8.3.0 to create the artwork and illustrations.

## 3. Results

### 3.1. Determination of the Optimal Dose of Iohexol

**Figure 2:**
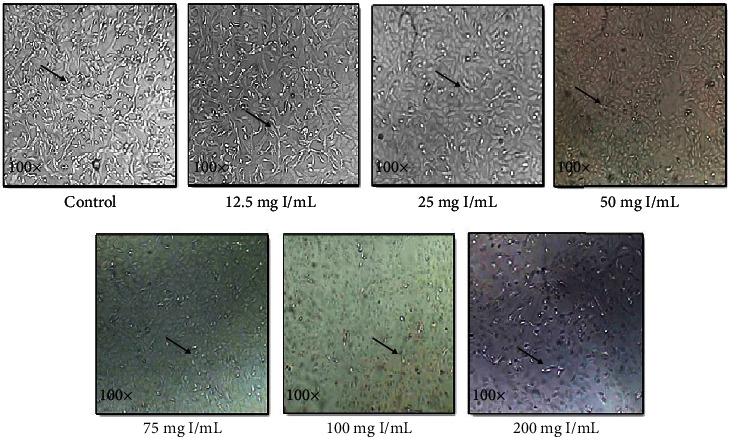
Vero cells under the microscope after subjecting to different doses of iohexol containing increasing concentration of iodine (I) (*n* = 4). Magnification 100x. The control group displays healthy, growing cells with elongated morphology (black arrows), whereas the cells incubated with iohexol show signs of rounding up.

Vero cells incubated with different concentrations of iohexol were examined under a microscope. 100% confluency was observed in the control cells which retained their primary elongated morphology. With a progressive increase in the concentration of iohexol, there was a concomitant increase in cell death. At a concentration of 50 mg iodine per mL of iohexol, nearly 50% cell lysis was evident with the maximum lysis occurring at 200 mg iodine per mL of iohexol concentration (Figure 2).

Vero cells were further exposed to increasing doses of iohexol to determine cell viability using MTT assay. As illustrated in Figure 1, in the dose-response study, the effect of iohexol was noted to be concentration-dependent. A significant decrease in cell viability was conspicuous starting from a concentration of 50 mg iodine per mL of iohexol with respect to the control (100% viable cells). Cell survival was the lowest at the maximum iohexol concentration of 200 mg iodine per mL (Figure 1). An effective dose of 100 mg iodine per mL of iohexol was used for the successive experiments.

### 3.2. Total Cellular Iron

**Figure 3:**
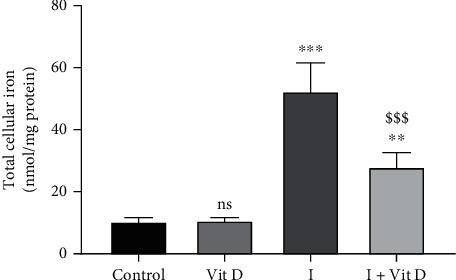
Relative concentrations of total cellular iron determined by atomic absorption spectroscopy in Vero cells treated with iohexol at a concentration of 100 mg iodine/mL or pretreated with 1 *μ*M vitamin D3. Results are represented as mean ± SD. One-way ANOVA and Tukey posttest (*n* = 4) were used to analyse the results. ns: not significant; *p* < 0.05, ^∗∗^*p* < 0.01, and ^∗∗∗^*p* < 0.001 (2-tailed) in comparison with the control; ^$^*p* < 0.05, ^$$^*p* < 0.01, and ^$$$^*p* < 0.001 (2-tailed) iohexol (I) versus iohexol+vitamin D (I+Vit D) groups.

Iohexol exposure caused a significant increase in the total iron content in the Vero cells (52.4 ± 9.1 nmol/mg protein, *p* < 0.001) compared with the control cells (10.4 ± 1.2 nmol/mg protein) and the cells incubated with vitamin D alone (10.8 ± 1.0 nmol/mg protein). The iron concentration was, however, noted to be significantly reduced in the cells pretreated with vitamin D (28.0 ± 4.7 nmol/mg protein) (Table 1, Figure 3).

### 3.3. Reactive Oxygen Species Production

**Figure 4:**
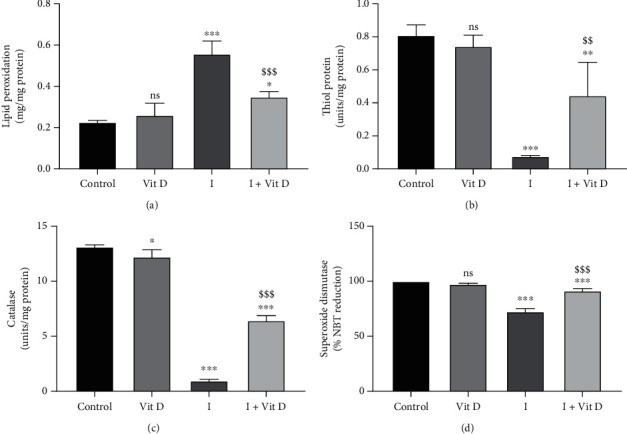
Levels of (a) lipid peroxidation, (b) thiol protein, (c) catalase, and (d) superoxide dismutase activity in Vero cells treated with iohexol at a concentration of 100 mg iodine/mL or pretreated with 1 *μ*M vitamin D3. Results are represented as mean ± SD. One-way ANOVA and Tukey posttest (*n* = 4) were used to analyse the results. ns: not significant; ^∗^*p* < 0.05, ^∗∗^*p* < 0.01, and ^∗∗∗^*p* < 0.001 (2-tailed) versus control; ^$^*p* < 0.05, ^$$^*p* < 0.01, and ^$$$^*p* < 0.001 (2-tailed) iohexol (I) versus iohexol+vitamin D (I+Vit D) groups.

Similar conditions were reproduced to assess the oxidative stress markers in Vero cells (Figure 4). There was a considerable increase in lipid peroxidation in cells exposed to iohexol alone (0.6 ± 0.1 mg/mg protein, *p* < 0.001) compared with the control cells (0.2 ± 0.0 mg/mg protein). Furthermore, a concomitant reduction in the antioxidant levels, namely, total thiol protein (0.5 ± 0.2 units/mg protein, *p* < 0.01), catalase (6.4 ± 0.4 units/mg protein, *p* < 0.001), and superoxide dismutase (91.4 ± 1.7 units/mg protein, *p* < 0.001), was observed in those cells. On the other hand, these changes were significantly reversed when the cells were treated with vitamin D prior to incubation with iohexol (Figure 4, Table 1). The cells treated with both vitamin D and iohexol displayed a relatively decreased oxidative stress than when treated with iohexol alone. Similarly, not much difference in these levels existed between the control cells and the cells exposed to calcitriol alone (Table 1, Figure 4).

### 3.4. Cell Toxicity Test Using DAPI Staining

**Figure 5:**
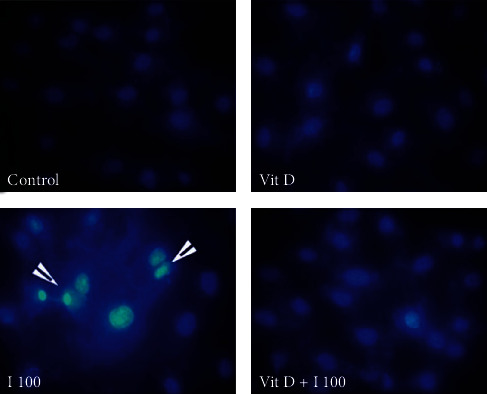
Fluorescent micrographs of Vero cells stained with DAPI (4′,6-diamidino-2-phenylindole) demonstrating more prominent nuclear condensation (shown in blue) in the iohexol-treated (I100) group (white arrows) compared to the iohexol+vitamin D-treated (Vit D+I100) group. Magnification 100x.

Vero cells treated with iohexol alone and after pretreatment with vitamin D3 were analysed after staining with DAPI. The morphology of the cells was investigated under a fluorescent microscope. The dead cells displayed a distorted appearance with fragmented or condensed nuclei (Figure 5).

### 3.5. Detection of Cell Viability by MTT Assay

**Figure 6:**
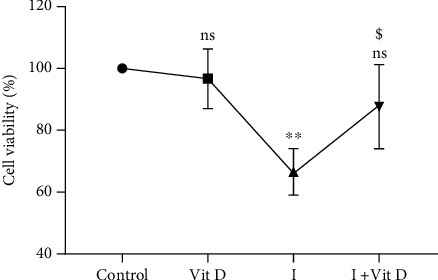
MTT 3-(4,5-dimethyl (thiazol-2-yl)-2,5-diphenyl tetrazolium bromide) assay to quantify cell viability in Vero cells exposed to iohexol and vitamin D3. Results are represented as mean ± SD. One-way ANOVA and Tukey posttest (*n* = 4) were used to analyse the results. ns: not significant; ^∗^*p* < 0.05, ^∗∗^*p* < 0.01, and ^∗∗∗^*p* < 0.001 (2-tailed) in comparison with the control; ^$^*p* < 0.05, ^$$^*p* < 0.01, and ^$$$^*p* < 0.001 (2-tailed) iohexol (I) versus iohexol+vitamin D (I+Vit D) groups.

Compared with the control group, cell viability was noted to decrease dramatically in iohexol-treated cells (*p* < 0.001) in a concentration-dependent manner. This was indicated by the lower absorbance rates of the experimental samples compared with negative control on MTT assay. However, prior exposure to vitamin D appeared to counteract the reduction in cell viability (*p* < 0.05) (Figure 6).

### 3.6. Correlation of Iron with Oxidative Stress and Cell Viability

**Figure 7:**
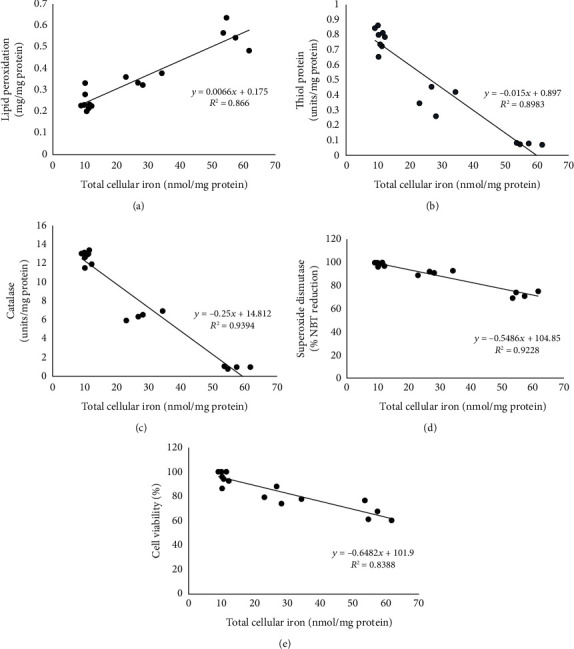
Scatter plot depicting the correlation between total cellular iron, oxidative stress, and cell viability in cells treated with iohexol: (a) lipid peroxidation, (b) thiol protein, (c) catalase, (d) superoxide dismutase activity, and (e) cell viability. NBT: nitroblue tetrazolium.

Total cellular iron showed a positive correlation with lipid peroxidation (Figure 7(a)) and a strong negative linear relationship with the antioxidants, namely, thiol protein, catalase, and superoxide dismutase (Figures 7(b)–7(d)) and with cell viability (Figure 7(e)).

## 4. Discussion

Although iron is an essential element, its ability to switch between ferrous and ferric states can be deleterious to renal cells. Renal cells possess highly regulated mechanisms to control cellular iron levels. Dysregulation of these mechanisms leads to disrupted cellular iron trafficking and consequent iron accumulation and altered iron homeostasis. Accordingly, suitable adaptive mechanisms are activated to enable the renal cells to remain viable. Here, we showed that calcitriol exerted a protective role against ferrotoxicity induced by iohexol in Vero cells. In the iohexol-treated cells, iron-mediated oxidative stress and a decrease in cell viability were reversed in the cells exposed to calcitriol.

This study exhibited a significant elevation in the total iron content in the Vero cells following exposure to the optimal dose of iohexol (Table 1, Figures 1–3). This was accompanied by increased oxidative stress and a significant decline in the antioxidant concentrations including those of the thiol protein, catalase, and superoxide dismutase (Table 1, Figure 4). In addition, significant cell death was visible on DAPI staining (Figure 5) along with a remarkable decrease in cell viability in a concentration-dependent manner (Figure 6). Strikingly, there existed a meaningful correlation between iron, oxidative stress, and cell viability (Figure 7). These findings are consistent with those of other studies in which iron and iron-containing proteins were revealed to cause direct injury to renal tubular cells *in vivo* using the whole kidney [66–68] and isolated proximal renal tubules [69, 70] and in other *in vitro* studies [71–74]. Similarly, García-Alfonso et al. proved ferrous [Fe(II)] and ferric [Fe(III)] forms of iron to produce a dose-dependent toxicity in Vero cells [75]. Iron-induced oxidative stress and cell death (ferroptosis) were also demonstrated in some rat and murine experimental AKI [20, 76–81]. Further, there occurred an increase in the kidney and urinary iron content in both animal models of AKI [82–85] and in humans [86–89]. Interestingly, the use of iron chelators was shown to reduce lipid peroxidation and improve renal functions [74, 90–93].

Most mammalian cells are protected from the detrimental effects of oxidant stress by tight control of iron homeostasis involving the processes of iron uptake, utilization, and storage. For instance, iron is transported in the circulation bound to transferrin [94] and sequestered inside the cell by ferritin [95] while hepcidin regulates the intake of iron and its distribution by binding to the basolateral membrane ferroportin, an iron exporter and facilitating its internalization and degradation [96–98]. As a result, very minute quantities of highly reactive and toxic free or labile iron are present in the circulation [21, 26, 99]. On the other hand, antioxidant renoprotective mechanisms also exist inside the kidney cells including superoxide dismutase and catalase to prevent oxidative injury [28]. Noteworthily, owing to its antioxidative property, vitamin D has been shown to provide cytoprotection [29–31].

This study noticed that cells treated with both calcitriol and iohexol tended to have decreased levels of cellular iron as well as a reduction in oxidative stress together with a concomitant elevation in the concentrations of antioxidants, namely, the thiol protein, catalase, and superoxide dismutase, in comparison with those cells incubated with iohexol alone. As vitamin D is known to be cytoprotective, one can speculate that the Vero cells incubated without 1,25-dihydroxyvitamin D3 were protected from the oxidative damage induced by iron.

This was supported by compelling evidence of renoprotection exerted by calcitriol and its analogues in several experimental cell cultures and animal models. While some studies have reported a reduction in malondialdehyde levels by vitamin D [100] [101], others have illustrated an increase in the antioxidants like glutathione [102]. In an *in vitro* study by Weih et al., calcitriol was noted to inhibit the proliferation of opossum kidney (OK) cells [103]. Paricalcitol, the synthetic vitamin D analogue, was elucidated by Ari et al. [100] to circumvent contrast-induced nephropathy by reducing oxidative stress. By virtue of its antioxidant effects, vitamin D was found to protect against AKI induced by aminoglycoside [102, 104], cyclosporine [105], rhabdomyolysis [106], and lipopolysaccharide [107]. Moreover, deficient calcitriol levels appeared to exacerbate inflammatory responses in rats with ischemia reperfusion injury [108]. Low vitamin D levels were also found to increase the risk of AKI and to predict mortality in critically ill patients [109, 110], denoting the significance of maintaining adequate vitamin D levels.

**Figure 8:**
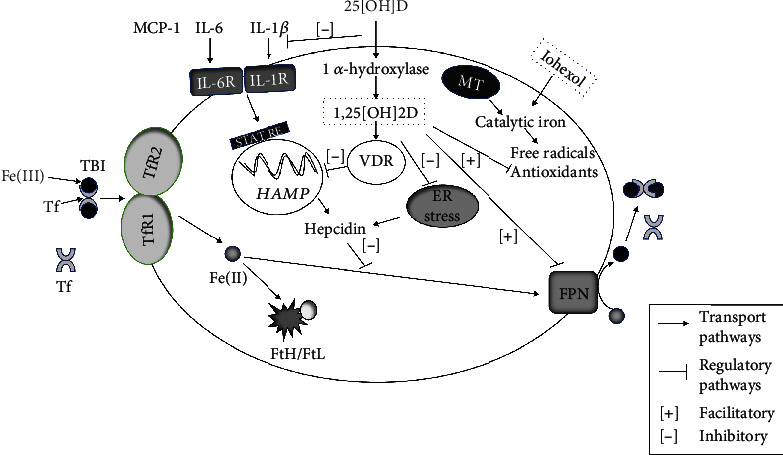
Mechanistic diagram illustrating the renoprotective role of calcitriol in iohexol-induced AKI. Iohexol increases the intracellular release of catalytic iron, for example, from mitochondria (MT) during the process of renal tissue injury. Concurrently, 1 *α*-hydroxylase (CYPB21) catalyses the conversion of 25-hydroxyvitamin D3 (25[OH]D) to active 1,25-dihydroxyvitamin D3 (I,25[OH]2D3), also known as calcitriol. Calcitriol binds to vitamin D receptor (VDR) which then binds to the proximal promoter region of HAMP gene containing vitamin D-responsive elements (VDREs) leading to suppression of HAMP gene and therefore hepcidin protein expression directly [112]. Vitamin D also indirectly reduces prohepcidin inflammatory cytokines, IL-6 and IL-1*β* [113] and MCP-1 [114]. Furthermore, vitamin D possesses antioxidant property and it relieves endoplasmic reticulum stress which is an inducer of hepcidin expression that could result in dysregulated cellular iron homeostasis [115]. Transferrin-bound iron (TBI) enters the cytosol through transferrin receptor protein (TfR1 and TfR2) and is either oxidized by ferritin heavy chain (FtH) to be stored intracellularly in the ferritin complex consisting of ferritin heavy and light chains (FtH/FtL) or is exported out of the cell by ferroportin (FPN). Hepcidin, the key regulator of systemic and cellular iron homeostasis, facilitates internalization and degradation of ferroportin. 25[OH]D: 25-hydoxyvitamin D3; 1,25[OH]2D: 1,25-dihyroxyvitamin D3; VDR: vitamin D receptor; MCP-1: monocyte chemoattractant protein 1; IL-6: interleukin 6; IL-1*β*: interleukin 1*β*; IL-6R: interleukin 6 receptor; IL-1*β*R: interleukin 1*β* receptor; STAT RE: signal transducer and activator of transcription responsive element; HAMP: hepcidin antimicrobial peptide; ER stress: endoplasmic reticulum stress; MT: mitochondria; Fe(III): ferric iron; Tf: transferrin; TBI: transferrin-bound iron; TfR1: transferrin receptor protein 1; TfR2: transferrin receptor protein 2; Fe(II): ferrous iron; FtH/FtL: ferritin heavy chain, ferritin light chain; FPN: ferroportin.

As described earlier, although contrast-induced acute kidney injury results from numerous pathophysiological processes such as ischemia due to changes in renal blood flow, direct renal tubular toxicity, and intratubular obstruction by the contrast material [8, 9], it would be helpful to analyse individual mechanisms independently *in vitro* utilizing a highly controlled cell culture system without the confounding factors encountered *in vivo*. Investigating the role of iron-mediated cytotoxicity without the influence of tubular and prerenal factors would therefore be very informative. This study accomplishes this objective and is the first to highlight such a reduction in iron-induced oxidative stress and injury by vitamin D in Vero cells exposed to iohexol. Of note, this study is an extension of a similar work carried out in Wistar rats by us [111], thereby providing comprehensive insights in to the pathophysiology of iohexol-induced ferrotoxicity and its possible amelioration by vitamin D both *in vitro* and *in vivo*. This cell model also paves way for the generation of an assay for preclinical testing of novel radiocontrast agents as well as for developing strategies to devise safer molecules. For a better understanding, the possible link between vitamin D and iron pathophysiological mechanisms inside the renal cell in the context of iohexol-induced nephrotoxicity is schematically represented in Figure 8.

There are certain limitations to our study. Firstly, metal ions including iron exist in free and bound forms [116]. Our study determined the total cellular iron using atomic absorption spectroscopy. It would have been desirable to examine the role of free iron, also known as labile or catalytic iron [26], in the cells in order to investigate its biological role as well as ferrotoxicity in a more intricate manner. Nonetheless, we could illustrate an increase in the overall cellular iron concentration in the Vero cells following iohexol use and a corresponding reduction in its levels following exposure to vitamin D, which clearly signify the existence of iron dyshomeostasis in this *in vitro* model of CI-AKI.

Next, this study did not assess the impact of calcitriol and iron on other intracellular mediators of iron handling including ferritin, hepcidin, and ferroportin. Lastly, iron chelators have been proven efficacious in animals inflicted with AKI by decreasing the luminal or intracellular iron levels [90]. These were, however, not used in this study. Perhaps, their use to diminish iron concentration and the resultant oxidative stress would have seemed more informative when interpreted in correlation with the cytoprotective effect of vitamin D. Importantly, it has to be understood that the mere association between iron and vitamin D noted in this research work is not a compelling evidence per se to infer that vitamin D annihilates ferrotoxicity. Notwithstanding this shortcoming, the fact that calcitriol has several pleiotropic effects and supported by the ample evidence demonstrating the cytoprotection it offers in several models of AKI and also in an *in vivo* experiment performed by us using Wistar rats [111], it seems rational to corroborate the role of vitamin D in overcoming iron-induced renal cytotoxicity. Yet, it is imperative to have more conclusive proof in this respect by means of effective translational research.

There are few key recommendations for future investigations depending on the present study. It will be important to further characterize and better understand the regulatory links between calcitriol and iron, especially at the cellular level involving the ferritin and the hepcidin-ferroportin axis and the associated downstream pathways by way of in-depth exploration using *in vitro* and *in vivo* models. Treatment of AKI is challenging owing to the complex nature of its pathogenicity and currently is largely supportive with there being no panacea for its optimal treatment. Consequently, multitarget therapeutic strategies are likely to be more effective. In the light of this as well as based on our study results, it would be worthwhile to evaluate the desirable effects of calcitriol in relation to iron chelating agents to overcome iron-induced cellular injury, ideally in subjects with AKI due to varied aetiology and severity. Also, it is of paramount importance to translate the findings of this cell culture study to patients by formulating treatment protocols following further validation through well-designed, randomized controlled clinical trials.

## 5. Conclusions

Our study findings further substantiate the crucial role played by iron in inducing renal cytotoxicity via oxidant stress in this model of iohexol-induced nephrotoxicity. Pretreatment with calcitriol protects the cells by significantly diminishing oxidative stress and ferrotoxicity and thereby enhancing cell viability. Overall, this work attempts to demonstrate an association between iron and vitamin D in the pathophysiology of AKI and provides some useful insights on iron being a vital target in the amelioration of nephrotoxicity, specifically by employing strategies to eliminate luminal or intracellular iron in addition to timely administration of vitamin D.

## Figures and Tables

**Table 1:** Descriptive statistics.

	Control (*n* = 4)	Vitamin D (*n* = 4)	Iohexol (*n* = 4)	Iohexol+vitamin D (*n* = 4)
Total cellular iron (nmol/mg protein)	10.4 ± 1.2	10.8 ± 1.0	52.4 ± 9.1^∗∗∗^	28.0 ± 4.7^∗∗^^$$$^
Lipid peroxidation (mg/mg protein)	0.2 ± 0.0	0.3 ± 0.1	0.6 ± 0.1^∗∗∗^	0.4 ± 0.0^∗^^$$$^
Thiol protein (units/mg protein)	0.8 ± 0.1	0.7 ± 0.1	0.1 ± 0.0^∗∗∗^	0.5 ± 0.2^∗∗^^$$^
Catalase (units/mg protein)	13.1 ± 0.2	12.2 ± 0.6^∗^	1.0 ± 0.1^∗∗∗^	6.4 ± 0.4^∗∗∗^^$$$^
Superoxide dismutase (units/mg protein)	100.0 ± 0.0	97.4 ± 0.8	72.4 ± 2.7^∗∗∗^	91.43 ± 1.7^∗∗∗^^$$$^
Cell viability (%)	100.0 ± 0.0	96.7 ± 9.6	66.6 ± 7.5^∗∗^	87.6 ± 13.6^$^

Results were represented as mean ± SD. One-way ANOVA and Tukey posttest (*n* = 4) were used to analyse the results. *p* < 0.05, ^∗∗^*p* < 0.01, and ^∗∗∗^*p* < 0.001 (2-tailed) versus control; ^$^*p* < 0.05; ^$$^*p* < 0.01, and ^$$$^*p* < 0.001 (2-tailed) iohexol versus iohexol+vitamin D groups.

## Data Availability

Data shall be available from the corresponding author upon request.

## References

[1] Li P. K. T., Burdmann E. A., Mehta R. L., Martin S. (2013). Acute kidney injury: global health alert. *Journal of Nephropathology*.

[2] Lakhmir S., Chawla, on behalf of the Acute Disease Quality Initiative Workgroup 16, Bellomo R. (2017). Acute kidney disease and renal recovery: consensus report of the acute disease quality initiative (ADQI) 16 workgroup. *Nature Reviews Nephrology*.

[3] Kinsey G. R., Okusa M. D. (2011). Pathogenesis of acute kidney injury: foundation for clinical practice. *American Journal of Kidney Diseases: The Official Journal of the National Kidney Foundation*.

[4] Bonventre J. V. (2010). Pathophysiology of AKI: injury and normal and abnormal repair. *Contributions to Nephrology*.

[5] Bagshaw S. M., Wald R. (2016). Acute kidney injury: timing of renal replacement therapy in AKI. *Nature Reviews Nephrology*.

[6] Morcos R., Kucharik M., Bansal P. (2019). Contrast-induced acute kidney injury: review and practical update. *Clinical Medicine Insights: Cardiology*.

[7] Rear R., Bell R. M., Hausenloy D. J. (2016). Contrast-induced nephropathy following angiography and cardiac interventions. *Heart (British Cardiac Society)*.

[8] Seeliger E., Sendeski M., Rihal C. S., Persson P. B. (2012). Contrast-induced kidney injury: mechanisms, risk factors, and prevention. *European Heart Journal*.

[9] McCullough P. A., Stacul F., Davidson C. (2006). Contrast-induced nephropathy: clinical insights and practical guidance - a report from the CIN consensus working panel-overview. *American Journal of Cardiology*.

[10] Heyman S. N., Rosen S., Rosenberger C. (2008). Renal parenchymal hypoxia, hypoxia adaptation, and the pathogenesis of radiocontrast nephropathy. *Clinical Journal of the American Society of Nephrology*.

[11] Heyman S. N., Rosen S., Khamaisi M., Idée J. M., Rosenberger C. (2010). Reactive oxygen species and the pathogenesis of radiocontrast-induced nephropathy. *Investigative Radiology*.

[12] Tumlin J., Stacul F., Adam A. (2006). Pathophysiology of contrast-induced nephropathy. *The American Journal of Cardiology*.

[13] Sendeski M. M. (2011). Pathophysiology of renal tissue damage by iodinated contrast media. *Clinical and Experimental Pharmacology & Physiology*.

[14] Sendeski M., Patzak A., Persson P. B. (2010). Constriction of the vasa recta, the vessels supplying the area at risk for acute kidney injury, by four different iodinated contrast media, evaluating ionic, nonionic, monomeric and dimeric agents. *Investigative Radiology*.

[15] Sendeski M. M., Persson A. B., Liu Z. Z. (2012). Iodinated contrast media cause endothelial damage leading to vasoconstriction of human and rat vasa recta. *American Journal of Physiology-Renal Physiology*.

[16] Walker V. J., Agarwal A. (2016). Targeting iron homeostasis in acute kidney injury. *Seminars in Nephrology*.

[17] Leaf D. E., Rajapurkar M., Lele S. S. (2015). Increased plasma catalytic iron in patients may mediate acute kidney injury and death following cardiac surgery. *Kidney International*.

[18] Lele S. S., Mukhopadhyay B. N., Mardikar M. M. (2013). Impact of catalytic iron on mortality in patients with acute coronary syndrome exposed to iodinated radiocontrast--The Iscom Study. *American Heart Journal*.

[19] Shah S. V., Rajapurkar M. M., Baliga R. (2011). The role of catalytic iron in acute kidney injury. *Clinical journal of the American Society of Nephrology : CJASN*.

[20] Baliga R., Ueda N., Shah S. V. (1993). Increase in bleomycin-detectable iron in ischaemia/reperfusion injury to rat kidneys. *The Biochemical Journal*.

[21] Zhao N., Enns C. A. (2012). Iron transport machinery of human cells. *Current Topics in Membranes*.

[22] Swaminathan S. (2018). Iron homeostasis pathways as therapeutic targets in acute kidney injury. *Nephron*.

[23] van Swelm R. P. L., JFM W. (2020). The multifaceted role of iron in renal health and disease. *Nature Reviews Nephrology*.

[24] Martines A. M. F., Masereeuw R., Tjalsma H., Hoenderop J. G., Wetzels J. F. M., Swinkels D. W. (2013). Iron metabolism in the pathogenesis of iron-induced kidney injury. *Nature Reviews Nephrology*.

[25] Ponka P. (1999). Cellular iron metabolism. *Kidney international Supplement*.

[26] Halliwell B., Gutteridge J. M. (1990). [1] Role of free radicals and catalytic metal ions in human disease: An overview. *Methods in Enzymology*.

[27] Basile D. P., Anderson M. D., Sutton T. A. (2012). Pathophysiology of acute kidney injury. *Comprehensive Physiology*.

[28] Perco P., Mayer G. (2018). Endogenous factors and mechanisms of renoprotection and renal repair. *European Journal of Clinical Investigation*.

[29] Lips P. (2006). Vitamin D physiology. *Progress in Biophysics and Molecular Biology*.

[30] Zhong W., Gu B., Gu Y., Groome L. J., Sun J., Wang Y. (2014). Activation of vitamin D receptor promotes VEGF and CuZn-SOD expression in endothelial cells. *The Journal of Steroid Biochemistry and Molecular Biology*.

[31] Sardar S., Chakraborty A., Chatterjee M. (1996). Comparative effectiveness of vitamin D3 and dietary vitamin E on peroxidation of lipids and enzymes of the hepatic antioxidant system in Sprague--Dawley rats. *International journal for vitamin and nutrition research Internationale Zeitschrift fur Vitamin- und Ernahrungsforschung Journal international de vitaminologie et de nutrition*.

[32] Medicine I., Board F. N., Calcium CRDRIVD (2011). *Dietary reference intakes for calcium and vitamin D*.

[33] Trang H. M., Cole D. E., Rubin L. A., Pierratos A., Siu S., Vieth R. (1998). Evidence that vitamin D3 increases serum 25-hydroxyvitamin D more efficiently than does vitamin D2. *The American Journal of Clinical Nutrition*.

[34] Armas L. A., Hollis B. W., Heaney R. P. (2004). Vitamin D2 is much less effective than vitamin D3 in humans. *The Journal of Clinical Endocrinology and Metabolism*.

[35] Holick M. F., Frommer J. E., McNeill S. C., Richtand N. M., Henley J. W., Potts JT Jr (1977). Photometabolism of 7-dehydrocholesterol to previtamin D3 in skin. *Biochemical and Biophysical Research Communications*.

[36] OKANO T., YASUMURA M., MIZUNO K., KOBAYASHI T. (1977). Photochemical conversion of 7-dehydrocholesterol into vitamin D3 in rat skins. *Journal of Nutritional Science and Vitaminology*.

[37] Cheng J. B., Levine M. A., Bell N. H., Mangelsdorf D. J., Russell D. W. (2004). Genetic evidence that the human CYP2R1 enzyme is a key vitamin D 25-hydroxylase. *Proceedings of the National Academy of Sciences of the United States of America*.

[38] Nykjaer A., Dragun D., Walther D. (1999). An endocytic pathway essential for renal uptake and activation of the steroid 25-(OH) vitamin D_3_. *Cell*.

[39] Takemoto F., Shinki T., Yokoyama K. (2003). Gene expression of vitamin D hydroxylase and megalin in the remnant kidney of nephrectomized rats. *Kidney International*.

[40] Fraser D. R., Kodicek E. (1970). Unique biosynthesis by kidney of a biological active vitamin D metabolite. *Nature*.

[41] Takeyama K., Kato S. (2014). The vitamin D3 1alpha-hydroxylase gene and its regulation by active vitamin D3. *Bioscience, Biotechnology, and Biochemistry*.

[42] Zehnder D., Hewison M. (1999). The renal function of 25-hydroxyvitamin D3-1alpha-hydroxylase. *Molecular and Cellular Endocrinology*.

[43] Bouillon R., Okamura W. H., Norman A. W. (1995). Structure-function relationships in the vitamin D endocrine system. *Endocrine Reviews*.

[44] Bland R., Zehnder D., Hewison M. (2000). Expression of 25-hydroxyvitamin D3-1alpha-hydroxylase along the nephron: new insights into renal vitamin D metabolism. *Current Opinion in Nephrology and Hypertension*.

[45] Haussler M. R., Whitfield G. K., Haussler C. A. (1998). The nuclear vitamin D receptor: biological and molecular regulatory properties revealed. *Journal of Bone and Mineral Research: the Official Journal of the American Society for Bone and Mineral Research*.

[46] Walters M. R. (1992). Newly identified actions of the vitamin D endocrine system. *Endocrine Reviews*.

[47] Bouillon R., Bischoff-Ferrari H., Willett W. (2008). Vitamin D and health: perspectives from mice and man. *Journal of Bone and Mineral Research: the Official Journal of the American Society for Bone and Mineral Research*.

[48] Nagpal S., Na S., Rathnachalam R. (2005). Noncalcemic actions of vitamin D receptor ligands. *Endocrine Reviews*.

[49] Feldman D., Krishnan A. V., Swami S., Giovannucci E., Feldman B. J. (2014). The role of vitamin D in reducing cancer risk and progression. *Nature Reviews Cancer*.

[50] Liu W.-C., Wu C.-C., Hung Y.-M. (2016). Pleiotropic effects of vitamin D in chronic kidney disease. *Clinica chimica acta*.

[51] Hill S. J., Fisher A. S., Lindon J. C., Tranter G. E., Koppenaal D. W. (2017). Atomic absorption, methods and instrumentation. *Encyclopedia of Spectroscopy and Spectrometry*.

[52] Sawyer D. T., Heineman W. R., Beebe J. M. (1984). *Chemistry Experiments for Instrumental Methods*.

[53] Skoog D. A., West D. M., Holler F. J. (2013). *Fundamentals of Analytical Chemistry*.

[54] Torre M., González M. C., Jiménez O., Rodríguez A. R. (1990). Study of analytical methods for iron determination in complex organic liquids by atomic absorption spectrometry. *Analytical Letters*.

[55] Ohkawa H., Ohishi N., Yagi K. (1979). Assay for lipid peroxides in animal tissues by thiobarbituric acid reaction. *Analytical Biochemistry*.

[56] Ellman G. L. (1959). Tissue sulfhydryl groups. *Archives of Biochemistry and Biophysics*.

[57] Sinha A. K. (1972). Colorimetric assay of catalase. *Analytical Biochemistry*.

[58] Winterbourn C. C., Hawkins R. E., Brian M., Carrell R. W. (1975). The estimation of red cell superoxide dismutase activity. *The Journal of Laboratory and Clinical Medicine*.

[59] Kapuscinski J. (2009). DAPI: a DNA-specific fluorescent probe. *Biotechnic & histochemistry: official publication of the Biological Stain Commission*.

[60] Kim K. D., Iwasaki O., Noma K., Marmorstein R. (2016). Chapter eight-an IF–FISH approach for covisualization of gene loci and nuclear architecture in fission yeast. *Methods in Enzymology*.

[61] Denizot F., Lang R. (1986). Rapid colorimetric assay for cell growth and survival: modifications to the tetrazolium dye procedure giving improved sensitivity and reliability. *Journal of Immunological Methods*.

[62] Mosmann T. (1983). Rapid colorimetric assay for cellular growth and survival: application to proliferation and cytotoxicity assays. *Journal of Immunological Methods*.

[63] Slater T. F., Sawyer B., Straeuli U. (1963). STUDIES ON SUCCINATE-TETRAZOLIUM REDUCTASE SYSTEMS III. *Biochimica et Biophysica Acta*.

[64] Price P., McMillan T. J. (1990). Use of the tetrazolium assay in measuring the response of human tumor cells to ionizing radiation. *Cancer Research*.

[65] Seth R., Yang C., Kaushal V., Shah S. V., Kaushal G. P. (2005). p53-dependent caspase-2 activation in mitochondrial release of apoptosis-inducing factor and its role in renal tubular epithelial cell injury. *The Journal of Biological Chemistry*.

[66] Hamazaki S., Okada S., Ebina Y., Fujioka M., Midorikawa O. (1986). Nephrotoxicity of ferric nitrilotriacetate. An electron-microscopic and metabolic study. *The American Journal of Pathology*.

[67] Nath K. A. (2006). Heme oxygenase-1: a provenance for cytoprotective pathways in the kidney and other tissues. *Kidney International*.

[68] Kovtunovych G., Eckhaus M. A., Ghosh M. C., Ollivierre-Wilson H., Rouault T. A. (2010). Dysfunction of the heme recycling system in heme oxygenase 1-deficient mice: effects on macrophage viability and tissue iron distribution. *Blood*.

[69] Zager R. A., Foerder C. A. (1992). Effects of inorganic iron and myoglobin on in vitro proximal tubular lipid peroxidation and cytotoxicity. *The Journal of Clinical Investigation*.

[70] Chen L., Zhang B. H., Harris D. C. (1998). Evidence suggesting that nitric oxide mediates iron-induced toxicity in cultured proximal tubule cells. *The American Journal of Physiology*.

[71] Sponsel H. T., Alfrey A. C., Hammond W. S., Durr J. A., Ray C., Anderson R. J. (1996). Effect of iron on renal tubular epithelial cells. *Kidney International*.

[72] Sheerin N. S., Sacks S. H., Fogazzi G. B. (1999). In vitro erythrophagocytosis by renal tubular cells and tubular toxicity by haemoglobin and iron. *Nephrology, dialysis, transplantation: official publication of the European Dialysis and Transplant Association-European Renal Association*.

[73] Zager R. A., Burkhart K. (1997). Myoglobin toxicity in proximal human kidney cells: roles of Fe, Ca2+, H2O2, and terminal mitochondrial electron transport. *Kidney International*.

[74] Paller M. S., Hedlund B. E. (1994). Extracellular iron chelators protect kidney cells from hypoxia/reoxygenation. *Free Radical Biology & Medicine*.

[75] García-Alfonso C., López-Barea J., Sanz P., Repetto G., Repetto M. (1996). Changes in antioxidative activities induced by Fe (II) and Fe (III) in cultured Vero cells. *Archives of Environmental Contamination and Toxicology*.

[76] Linkermann A., Skouta R., Himmerkus N. (2014). Synchronized renal tubular cell death involves ferroptosis. *Proceedings of the National Academy of Sciences of the United States of America*.

[77] Baliga R., Zhang Z., Baliga M., Shah S. V. (1996). Evidence for cytochrome P-450 as a source of catalytic iron in myoglobinuric acute renal failure. *Kidney International*.

[78] Baliga R., Zhang Z., Baliga M., Ueda N., Shah S. V. (1998). In vitro and in vivo evidence suggesting a role for iron in cisplatin-induced nephrotoxicity. *Kidney International*.

[79] Paller M. S. (1988). Hemoglobin- and myoglobin-induced acute renal failure in rats: role of iron in nephrotoxicity. *The American Journal of Physiology*.

[80] Kirschner R. E., Fantini G. A. (1994). Role of iron and oxygen-derived free radicals in ischemia-reperfusion injury. *Journal of the American College of Surgeons*.

[81] Shah S. V., Walker P. D. (1988). Evidence suggesting a role for hydroxyl radical in glycerol-induced acute renal failure. *The American Journal of Physiology*.

[82] Veuthey T., D'Anna M. C., Roque M. E. (2008). Role of the kidney in iron homeostasis: renal expression of prohepcidin, ferroportin, and DMT1 in anemic mice. *American Journal of Physiology. Renal Physiology*.

[83] Ueda N., Baliga R., Shah S. V. (1996). Role of ‘catalytic’ iron in an animal model of minimal change nephrotic syndrome. *Kidney International*.

[84] Zager R. A., Foerder C., Bredl C. (1991). The influence of mannitol on myoglobinuric acute renal failure: functional, biochemical, and morphological assessments. *J Am Soc Nephrol*.

[85] Harris D. C., Tay C., Nankivell B. J. (1994). Lysosomal iron accumulation and tubular damage in rat puromycin nephrosis and ageing. *Clinical and Experimental Pharmacology & Physiology*.

[86] Moreno J. A., Martín-Cleary C., Gutiérrez E. (2012). AKI associated with macroscopic glomerular hematuria: clinical and pathophysiologic consequences. *Clinical journal of the American Society of Nephrology: CJASN*.

[87] SEARS D. A. V. I. D. A., ANDERSON P. E. A. R. L. R., FOY A. R. T. H. U. R. L., WILLIAMS H. A. R. O. L. D. L., CROSBY W. I. L. L. I. A. M. H. (1966). Urinary iron excretion and renal metabolism of hemoglobin in hemolytic diseases. *Blood*.

[88] Ballarin J., Arce Y., Balcells R. T. (2011). Acute renal failure associated to paroxysmal nocturnal haemoglobinuria leads to intratubular haemosiderin accumulation and CD163 expression. *Nephrology Dialysis Transplantation*.

[89] Wang H., Nishiya K., Ito H., Hosokawa T., Hashimoto K., Moriki T. (2001). Iron deposition in renal biopsy specimens from patients with kidney diseases. *American Journal of Kidney Diseases: The Official Journal of the National Kidney Foundation*.

[90] Paller M. S., Hedlund B. E. (1988). Role of iron in postischemic renal injury in the rat. *Kidney International*.

[91] Mori K., Lee H. T., Rapoport D. (2005). Endocytic delivery of lipocalin-siderophore-iron complex rescues the kidney from ischemia-reperfusion injury. *The Journal of Clinical Investigation*.

[92] de Vries B., Walter S. J., von Bonsdorff L. (2004). Reduction of circulating redox-active iron by apotransferrin protects against renal ischemia-reperfusion injury. *Transplantation*.

[93] Mishra J., Mori K., Ma Q. (2004). Amelioration of ischemic acute renal injury by neutrophil gelatinase-associated lipocalin. *J Am Soc Nephrol*.

[94] Cazzola M., Huebers H. A., Sayers M. H., MacPhail A. P., Eng M., Finch C. A. (1985). Transferrin saturation, plasma iron turnover, and transferrin uptake in normal humans. *Blood*.

[95] Theil E. C. (2013). Ferritin: the protein nanocage and iron biomineral in health and in disease. *Inorganic Chemistry*.

[96] Drakesmith H., Nemeth E., Ganz T. (2015). Ironing out ferroportin. *Cell Metabolism*.

[97] Nemeth E., Tuttle M. S. (2004). Hepcidin regulates cellular iron efflux by binding to ferroportin and inducing its internalization. *Hepcidin regulates cellular iron efflux by binding to ferroportin and inducing its internalization. science*.

[98] Qiao B., Sugianto P., Fung E. (2012). Hepcidin-induced endocytosis of ferroportin is dependent on ferroportin ubiquitination. *Cell Metabolism*.

[99] Hentze M. W., Muckenthaler M. U., Galy B., Camaschella C. (2010). Two to tango: regulation of mammalian iron metabolism. *Cell*.

[100] Ari E., Kedrah A. E., Alahdab Y. (2012). Antioxidant and renoprotective effects of paricalcitol on experimental contrast-induced nephropathy model. *The British Journal of Radiology*.

[101] Husain K., Suarez E., Isidro A., Ferder L. (2010). Effects of paricalcitol and enalapril on atherosclerotic injury in mouse aortas. *American Journal of Nephrology*.

[102] Hur E., Garip A., Camyar A. (2013). The effects of vitamin d on gentamicin-induced acute kidney injury in experimental rat model. *International Journal of Endocrinology*.

[103] Weih M., Orth S., Weinreich T., Reichel H., Ritz E. (1994). Inhibition of growth by calcitriol in a proximal tubular cell line (OK). *Nephrology Dialysis Transplantation*.

[104] Bulut G., Basbugan Y., Ari E. (2016). Paricalcitol may improve oxidative DNA damage on experimental amikacin-induced nephrotoxicity model. *Renal Failure*.

[105] Park J. W., Bae E. H., Kim I. J. (2010). Paricalcitol attenuates cyclosporine-induced kidney injury in rats. *Kidney International*.

[106] Reis N. G., Francescato H. D. C., de Almeida L. F., da Silva C. G. A., Costa R. S., Coimbra T. M. (2019). Protective effect of calcitriol on rhabdomyolysis-induced acute kidney injury in rats. *Scientific Reports*.

[107] Du J., Jiang S., Hu Z. (2019). Vitamin D receptor activation protects against lipopolysaccharide-induced acute kidney injury through suppression of tubular cell apoptosis. *American Journal of Physiology-Renal Physiology*.

[108] de Bragança A. C., Volpini R. A., Canale D. (2015). Vitamin D deficiency aggravates ischemic acute kidney injury in rats. *Physiological Reports*.

[109] Lee P., Eisman J. A., Center J. R. (2009). Vitamin D deficiency in critically ill patients. *The New England Journal of Medicine*.

[110] Braun A. B., Litonjua A. A., Moromizato T., Gibbons F. K., Giovannucci E., Christopher K. B. (2012). Association of low serum 25-hydroxyvitamin D levels and acute kidney injury in the critically ill. *Critical Care Medicine*.

[111] Annamalai C., Ganesh R. N., Viswanathan P. (2020). Ferrotoxicity and its amelioration by endogenous vitamin D in experimental acute kidney injury. *Experimental Biology and Medicine*.

[112] Bacchetta J., Zaritsky J. J., Sea J. L. (2014). Suppression of iron-regulatory hepcidin by vitamin D. *J Am Soc Nephrol*.

[113] Zughaier S. M., Alvarez J. A., Sloan J. H., Konrad R. J., Tangpricha V. (2014). The role of vitamin D in regulating the iron-hepcidin-ferroportin axis in monocytes. *Journal of Clinical & Translational Endocrinology*.

[114] Alvarez J. A., Zughaier S. M., Law J. (2013). Effects of high-dose cholecalciferol on serum markers of inflammation and immunity in patients with early chronic kidney disease. *European Journal of Clinical Nutrition*.

[115] Riek A. E., Oh J., Sprague J. E. (2012). Vitamin D suppression of endoplasmic reticulum stress promotes an antiatherogenic monocyte/macrophage phenotype in type 2 diabetic patients. *The Journal of Biological Chemistry*.

[116] Dean K. M., Qin Y., Palmer A. E. (2012). Visualizing metal ions in cells: an overview of analytical techniques, approaches, and probes. *Biochimica et Biophysica Acta (BBA) - Molecular Cell Research*.

